# Catgut Implantation at Acupoint Reduces Immune Reaction in a Rat Model of Allergic Rhinitis

**DOI:** 10.1155/2018/7629239

**Published:** 2018-07-05

**Authors:** Shasha Yang, Jing Wu, Qinxiu Zhang, Xinrong Li, Daien Liu, Bin Zeng, Hongjiao Gao, Xiaolin Yan, Zhendong Zhong

**Affiliations:** ^1^Chengdu University of Traditional Chinese Medicine, Chengdu, Sichuan 610072, China; ^2^Guiyang College of Traditional Chinese Medicine, Guiyang, Guizhou 550002, China; ^3^Department of Otorhinolaryngology, Head and Neck Surgery of the Teaching Hospital of Chengdu University of Traditional Chinese Medicine, Chengdu, Sichuan 610072, China; ^4^Sichuan Academy of Medical Sciences and Sichuan Provincial People's Hospital, Chengdu 610072, China

## Abstract

Allergic rhinitis (AR), an IgE-mediated response, is characterized by a Th2-type immunological pattern together with mast cells activation. Acupuncture, with the use of implanted catgut, is a traditional therapy that has been widely applied for the treatment of AR. However, the exact mechanism of the immunomodulatory effects of catgut implantation at acupoint (CIAA) remains unclear, in part due to the lack of a suitable laboratory animal model. We developed and optimized a rat model of ovalbumin- (OVA-) induced allergic inflammation, characterized by increased IL-4, sIgE, and SP and reciprocal decrease of IFN-*γ*. In the present study, we have further used this model to address the immunomodulatory effects of CIAA stimulation at Yingxiang (LI20) and Zusanli (ST36) acupoints and to elucidate the mechanisms involved in the regulation of SP, sIgE, IL-4, IFN-*γ*, TLR2, and TLR4. After AR model was established via OVA challenge, the rats were randomized as follows: control, model, sham-operated, 1-week CIAA (C1), 2-week CIAA (C2), and Budesonide nasal spray. The C1 and C2 groups were subjected to the bilateral acupoint Yingxiang (LI20) and Zusanli (ST36), respectively. Multiple analyses and quantifications were performed, which revealed that due to the persistent stimulus to acupoints by embedding catgut, the C2 group improved AR symptoms, compared to the C1 group. We conclude that CIAA at the Yingxiang (LI20) and Zusanli (ST36) acupoints effectively reduces allergic symptoms and inflammatory parameters in the rat model of AR. Thus, CIAA treatment is potentially an alternative therapeutic modality in AR.

## 1. Introduction

Allergic rhinitis (AR) is an IgE-mediated response, characterized by Th2 immunological pattern, mainly occurring together with mast cells activation [1], thus inducing sneezing, itching, nasal congestion, watery rhinorrhea, and even impaired quality of life (QOL) [2–5]. AR is a highly prevalent chronic disease that affects approximately 40% of the world's population [6]. In China, a cross-population study, conducted in 11 major cities, indicated that the prevalence of AR ranges from 8.7% to 24.1% [7]. Although Intranasal corticosteroids and antihistamines exhibit clinical efficacy for the treatment of AR [8], they cause inevitable side effects such as hormone resistance, drowsiness, and sedation. Thus, finding an effective treatment of AR has remained a major challenge.

Acupuncture is a traditional therapy that has been widely applied for the treatment of AR. In 2015, acupuncture treatment was incorporated into the United States guidelines for the treatment of AR [9]. Acupuncture has been shown to have anti-inflammatory effects, including regulation of proinflammatory cytokines, proinflammatory neuropeptides, and neurotrophins [10, 11]. While CIAA is based on traditional acupuncture, persistent stimulus to acupoints by embedding catgut has been considered to extend its durative effect [12]. A systematic recent review on CIAA for AR indicated that CIAA is an effective therapy and has no adverse events [13]. Our previous studies have also confirmed the ameliorative effect of CIAA on AR symptoms in patients [14]. Previously, animal experiments also demonstrated that CIAA downregulated neurogenic inflammation, mainly induced by SP, CGRP (calcitonin gene-related peptide), and VIP (vasoactive intestinal peptide) [15].

AR is primarily an inflammatory IgE-mediated response [16], characterized by an enhanced Th2 immunological pattern (e.g., IL-4) and an inhibited Th1 pattern (such as IFN-*γ*), together with the activation of mast cells, goblet cells, and eosinophils. Since excessive activation of Th2 drives AR, it is crucial to regulate the balance between Th1 and Th2 [17] to reverse the established Th2 response and avoid AR.

Toll-like receptors (TLRs) are natural immune receptors that recognize a variety of pathogen-associated molecular patterns (such as DAMPs or PAMPs) and play an important role in the immune defense response of the nasal mucosa [18, 19]. TLRs are widely distributed in the B and T-lymphocytes, dendritic cells, and mast cells [20]. In addition to the PAMP, TLRs can further identify a large number of endogenous molecules derived from tissue damage [21], namely, stress cells, extracellular matrix degraded cytokines, and chemokines.

We have developed a rat model of ovalbumin- (OVA-) induced allergic inflammation, characterized by increased IL-4, sIgE and SP and reciprocal decrease of IFN-*γ*. In the present study, we have used this model to address the immunomodulatory effects of CIAA stimulation at Yingxiang (LI20) and Zusanli (ST36) acupoints and to elucidate the mechanisms involved in the regulation of SP, sIgE, IL-4, IFN-*γ*, TLR2, and TLR4. Our results provide a molecular foundation to the clinical applications of CIAA in treating AR.

## 2. Materials and Methods

### 2.1. Chemicals and Reagents

OVA (Sigma A8040, USA) was used as antigen, and aluminum hydroxide as adjuvant (lot No. 201110328, Chengdu Kelong Chemical Factory). The No. 9 needle (YZB/Su0313-2007, Yangzhou Co. Ltd., China), and the No. 000 catgut (YY1116-2002, Shanghai Co. Ltd., China) are disposable applications. sIgE, IL-4, and IFN-*γ* enzyme-linked immunosorbent assay (ELISA) kits were purchased from Abcam (Yonghui Bio Co. Ltd., Beijing, China). The SP and TLR2 polyclonal antibody (rabbit anti-mouse) and TLR4 monoclonal antibody (mouse anti-rat) were from Abcam (Trading Co. Ltd., Shang Hai, China). Sodium citrate buffer (0.01 M, pH 6.0) was prepared for dilution. Microscopic image acquisition and analysis system (MikeAudiBA200 Digital and Image-Pro Plus 6.0, USA) and ChemiDoc XRS gel imaging apparatus (Bio-Rad, USA) were purchased from the respective manufacturers.

### 2.2. Animal Preparation

Studies were carried out on adult male Sprague-Dawley rats (250-300g, 8-10 weeks of age), obtained from Da Shuo Biological Technology Co. Ltd., China, with certificate No. SCXK (Chengdu) 2015-030. Before the experiment began, all rats were adapted for 1 week in Experimental Animal Center of the Chengdu University of TCM. In general, all procedures with animals avoided and / or minimized discomfort, distress, and pain to the animals. The rats were randomly divided into six groups (n = 7 or 8 in each group): control, model, sham CIAA, CIAA for 1 week (C1), CIAA for 2 weeks (C2), and Budesonide-treated (by nasal spray).

### 2.3. Establishment of the AR Model

The AR model was established using an ovalbumin (OVA) sensitization method [15]. Rats were sensitized (days 1-13) with 7 intraperitoneal (i.p.) injections of 0.3 mg OVA (Sigma A8040, USA) as antigen and 30 mg aluminum hydroxide as adjuvant dissolved in 1 ml of saline. Upon finishing the i.p. immunizations, the nasal antigen challenge (days 14-21) was performed with intranasal dripping of 50 *μ*l of 2% OVA daily for 7 consecutive days. The animals in the control group were administered with the same volume of saline. All animals were closely observed for developing any nasal responses of sneezing, watery rhinorrhea, and scraping for 30 minutes after each challenge. Then the symptoms and signs of AR were provoked (days 22-24) with intranasal dripping of 80 *μ*l of 1% OVA daily for 3 consecutive days. Ten minutes after the last provocation with 1% OVA, all animals were subjected to i.p. injection of 1% sodium pentobarbital (50 mg/kg) to collect tail venous blood. Serum levels of cytokines were measured using enzyme-linked immunosorbent assay (ELISA). Specific IgE (sIgE), IL-4, and IFN-*γ* levels were measured to test whether AR model was successfully established. Finally, the sensitization was maintained (day 25-end) with intranasal dripping of 50 *μ*l of 1% OVA every other day. At day 40, final blood samples and nasal mucosa were collected for the various assays.

### 2.4. CIAA Treatment

The rats were placed in tailor-made mouse cages, so that their head and bilateral legs were sufficiently exposed. Stainless steel needles (No. 9) were bilaterally inserted at acupoint Yingxiang (LI20), 2-4 mm in depth, located at ~3 mm on both sides of the nostril, and the catgut was pushed quickly into acupoint in the C1 and C2 group. At the same time, needles were bilaterally inserted at acupoint Zusanli (ST36), ~2-4 mm in depth, located at 5 mm lateral and distal to the anterior tubercle of the tibia, and, as before, the catgut was pushed quickly into acupoint in the C1 and C2 group. All rats were conscious when CIAA was performed. In contrast to the CIAA groups, the sham group included sole acupuncture at point Yingxiang (LI20) and Zusanli (ST36) in the absence of catgut implantation. CIAA was conducted under sterile conditions. The tissue area was disinfected at the points with iodophor. Rats in the Budesonide group were intranasally administered with Budesonide (2.5 *μ*g/nasal cavity) daily for 14 consecutive days.

### 2.5. Assay of Rat Behavior

The numbers of sneezing and nose rubbing motions during 30 min after the final allergen challenge were recorded in each experimental group. Following superimposition of the recording results, a total score of >5 was used as benchmark for successful establishment of the AR [22].

### 2.6. Specimen Collection

The animals of all groups (n = 7 or 8) were sacrificed with i.p. injection of 3% sodium pentobarbital (30 mg/kg) and transcardially perfused with 350 mL 0.9% saline and fixed in a solution containing 2% paraformaldehyde and 1.25% glutaraldehyde phosphate buffer solution (pH = 7.2). Blood collected from the femoral artery was subjected to measurements of serum sIgE, IL-4, and INF-*γ*. Part of the nasal mucosa was quickly removed from the respiratory area of the nasal chamber and postfixed in 4% paraformaldehyde, dissolved in 0.1 M phosphate buffer. Another part of nasal mucosa was quickly frozen in liquid nitrogen. The tissues were sent for routine histological examination.

### 2.7. Degranulation Rate Assay of Mast Cells

Following the manufacturer's recommendations (toluidine blue, Biotech, USA), the degranulation rate of mast cells was detected by 1% toluidine blue staining, in which a total of 3 images for each sample were acquired and the number of degranulation of mast cells were recorded by microscopic examination. The degranulation rate (%) of mast cells was then calculated.

### 2.8. Enzyme-Linked Immunosorbent Assay (ELISA)

Serum levels of specific IgE (sIgE), IL-4, and IFN-*γ* were measured by solid-phase enzyme-linked immunosorbent assay (ELISA) in accordance with the manufacturer's instructions. Bound immunoglobulin isotypes were detected with specific secondary antibody. Biotin-conjugated rat anti-mouse IgE, IL-4, and IFN-*γ* antibodies were purchased from BD Pharmingen, Beijing, China.

### 2.9. Immunohistochemistry (IHC)

Paraffin sections of nasal mucosa tissue were stained with streptavidin-peroxidase method to examine SP expression. The sections were incubated in 3% hydrogen peroxide (H_2_O_2_)/methanol for 15 min. After washing three times in PBS (pH 7.2-7.4) for 5 min each, they were immersed in 0.01 M citrate buffer (pH 6.0) 5 min, followed by two washes with PBS. Nonspecific binding was blocked by incubating with normal goat serum for 20 min at 37°C. The sections were then incubated with rabbit anti-SP (1:200 dilution) overnight at 4°C and then with a biotinylated goat anti-rabbit IgG for 30 min. Following incubation with horseradish peroxidase- (HRP-) conjugated streptomycin ovalbumin reagent, the sections were colored using a concentrated DAB kit, and observation and acquisition of images were done in the mounting media. The immunopositive cells were detected using microscopic analysis (at 400x magnification), and the intensity of SP-positive expression was quantified by the average number of positively stained cells per field.

### 2.10. Immunofluorescence Analysis

The tissues, embedded in OCT and frozen in liquid nitrogen, were cut into serial 8 *μ*m sections using a cryostat (Leica CM 1900, Bensheim, Germany), placed on APES (3-aminopropyltriethoxysilane)-coated glass slides, dried at room temperature for 30 min and then stored in −80°C [23, 24]. Sections were blocked with 1% fetal bovine serum (dissolved in PBS, supplemented with 0.3% Triton X-100) for 1 h at room temperature. They were then incubated with antibodies against TLR2 and TLR4 in a humid chamber at 4°C overnight. After rinsing with 0.1 M PBS three times, the sections were incubated with secondary fluorescein-conjugated antibodies for 60 min at 37°C and then with DAPI (Beyotime 5 mg/ml, China) to stain the nuclei for 30 min at room temperature. Images were acquired using a confocal microscope (A1R; Nikon, Japan).

### 2.11. Immunoblot (Western Blot)

Cryopreserved tissues were thawed in a 37°C water bath and lysed by the addition of 2.5 mL of RIPA lysate (RIPA  :  cocktail = 100 : 1). Lysates were centrifuged at 12,000 rpm for 30 min at 4°C. The protein concentration of the cytosolic fraction was determined using a bicinchoninic acid (BCA) protein assay kit. The samples were boiled at 100°C in a sodium dodecyl sulfate (SDS) gel loading buffer for 10 min and loaded onto a 10% SDS polyacrylamide gel. The separated proteins were electrotransferred to a membrane, which were then incubated in 5% skim milk for 60 min at room temperature to block nonspecific binding. They were then incubated with rabbit antibodies against TLR2 (1:1000 dilution) or TLR4 (1:1000 dilution) overnight at 4°C. The membranes were also probed with a monoclonal antibody specific for actin (1:5000 dilution, MAB1501, Chemicon) as an internal control for the cytosolic fraction. After washing, membranes were incubated with HRP-linked anti-rabbit IgG (1:5000 dilution) and HRP-linked anti-mouse IgG (1:5000 dilution) antibody in PBS for 1h at RT. Proteins were detected using an enhanced chemiluminescence reagent kit. Densitometric analysis was performed using Quantity One software package. The TLR2/*β*-actin and TLR4/*β*-actin grayscale signal ratios were quantified.

### 2.12. Statistical Analysis

Data are presented as mean ± standard deviation (SD) (SPSS Statistical analysis software version 20.0). All variables indicated approximately normal distribution by Kolmogorov-Smirnov test and homogeneity by Levene's test, simultaneously one-way analysis of variance (ANOVA), followed by post hoc analysis using the Student-Newman-Keuls- (SNK-) q test. Differences were considered to be statistically significant at a P value < 0.05 (*P* < 0.05).

## 3. Results

### 3.1. Establishment of the AR Model

In order to evaluate whether the AR model was established in the animal, tail venous blood from 7-8 rats, belonging to model, sham, C1, C2, and Budesonide groups, were collected. Serum-specific IgE (sIgE), IL-4, and IFN-*γ* levels, compared to the control group, were used for assessment of AR. While the sIgE levels are strong diagnostic indicators, the levels of IL-4 and IFN-*γ*, respectively, reflect the Th2 and Th1 cell populations. Results are presented as mean ± SD, and* P*<0.05 or* P*<0.01 was considered statistically significant differences (Table 1).

In what follows, we have used this animal model to test the effects of CIAA on multiple biological and molecular parameters, relevant to allergic rhinitis (AR).

### 3.2. Effects of CIAA on Animal Behavior

**Figure 1:**
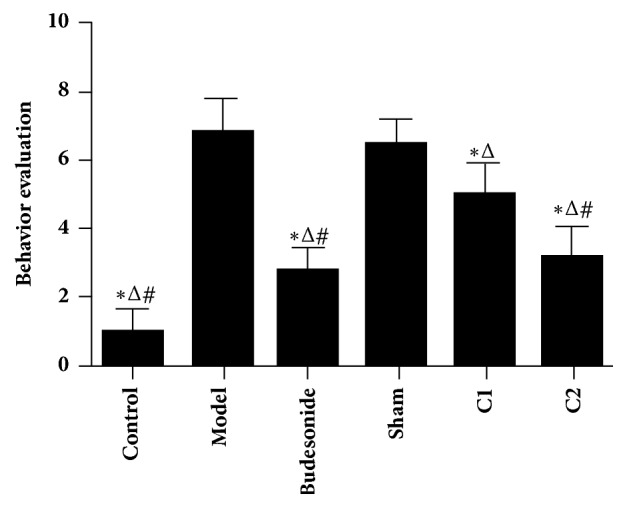
**Effect of CIAA on behavior evaluation.** The experimental protocol, including the administration of CIAA in rats, the various controls, and measurement of animal behavior have been detailed in Materials and Methods. *∗P *< 0.01 versus model group; ^△^*P *< 0.01 versus sham group; and ^#^*P *< 0.01 versus C1 group.

Behavior evaluation of each group of rat included studies of sneezing, scratching, nasal discharge, and foraging symptoms. The behavior scores showed no apparent difference between the model group and sham group (*P*>0.05). However, values of the C2 group were significantly lower than those of the model, sham and C1 groups (*P*<0.01). We also observed that the Budesonide group showed no significant difference compared to the C2 group (*P* >0.05, Figure 1).

### 3.3. Effects of CIAA on the Degranulation Rates of Mast Cells

**Figure 2:**
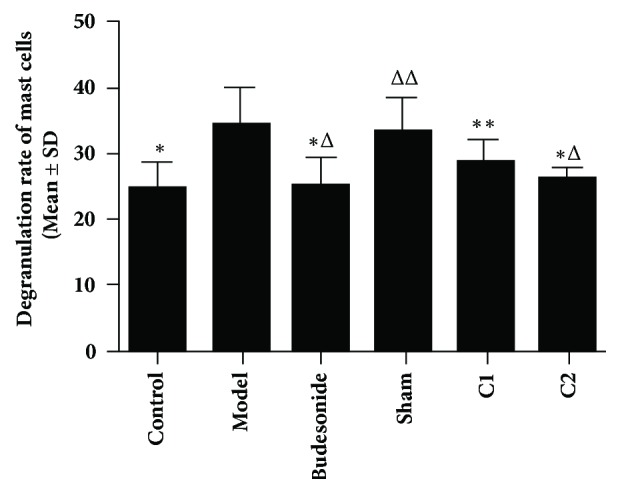
**Effects of CIAA on the degranulation rates of mast cells.** The experimental protocol (essentially the same as in Figure 1) and measurement of degranulation rates have been detailed in Materials and Methods. *∗P *< 0.01 versus the model group; *∗∗P *< 0.05 versus the model group; ^△^*P*<0.01 versus the sham group; ^△△^*P *< 0.05 versus the C1 group; C1 versus C2* P*>0.05, Budesonide versus C1/C2,* P *> 0.05.

The degranulation rates of mast cells were examined by microscopy. A total of 3 images for each sample were acquired and the number of degranulation of mast cells was counted and recorded. The degranulation rates of the mast cells showed no significant difference among the C1,C2 and Budesonide groups (*P*>0.05). However, the rates in the C2 and Budesonide groups were significantly lower than the model or sham groups (*P* < 0.01). The C1 group also showed a lower rate, compared to the model or sham group (*P*< 0.05; Figure 2).

### 3.4. Effects of CIAA on sIgE, IL-4, and IFN-*γ* Levels

**Figure 3:**
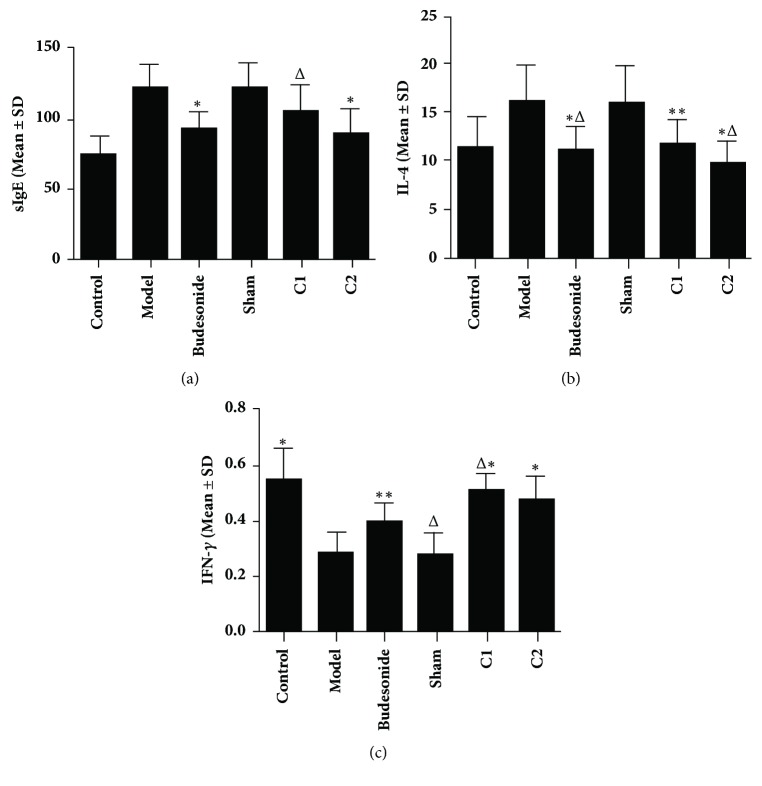
**Effects of CIAA on (a) sIgE, (b) IL-4, and (c) IFN-**γ** levels. **The experimental protocol (essentially the same as in Figure 1) and measurement of the three molecules have been detailed in Materials and Methods. (a) sIgE: *∗P *< 0.01 versus the model group; ^△^*P *< 0.05 versus the model group; C1 versus C2* P > *0.05; Budesonide versus C1/C2,* P > *0.05. (b) IL-4: *∗P *< 0.01 versus the model group; *∗∗P *< 0.05 versus the model group; ^△^*P *< 0.01 versus the sham group; C1 versus C2,* P*>0.05; Budesonide versus C1/C2,* P > *0.05; (c) IFN-*γ*: *∗P*<0.01 versus the model group; *∗∗P*<0.05 versus the model group; ^△^*P*<0.05 versus the Budesonide group; C1 versus C2,* P*>0.05; Budesonide versus C2,* P*>0.05.

In ELISA analysis (Figure 3), the serum IgE (sIgE) showed no significant difference among the C1, C2, and Budesonide groups (P>0.05). However, the sIgE was significantly lower in the C2 and Budesonide groups than that in the model and sham groups (P < 0.01; Figure 3(a)). Besides, the model group was similar to the sham group in sIgE levels. The serum IL-4 showed no significant difference among the C1, C2, and Budesonide groups (P > 0.05). However, the serum IL-4 was significantly lower in the C2 and Budesonide groups than in the model and sham groups (P < 0.01; Figure 3(b)), but the model and the sham groups were similar (P > 0.05). Lastly, the serum IFN-*γ* showed no significant difference between the C1, C2, and Budesonide groups (P>0.05). However, it was lower in the Budesonide group than in the C1, model, and sham groups (*P* < 0.05 and* P* < 0.01, Figure 3(c)), whereas the model and the sham groups were similar with no significant difference (*P *> 0.05).

### 3.5. Effect of CIAA on SP Expression

**Figure 4:**
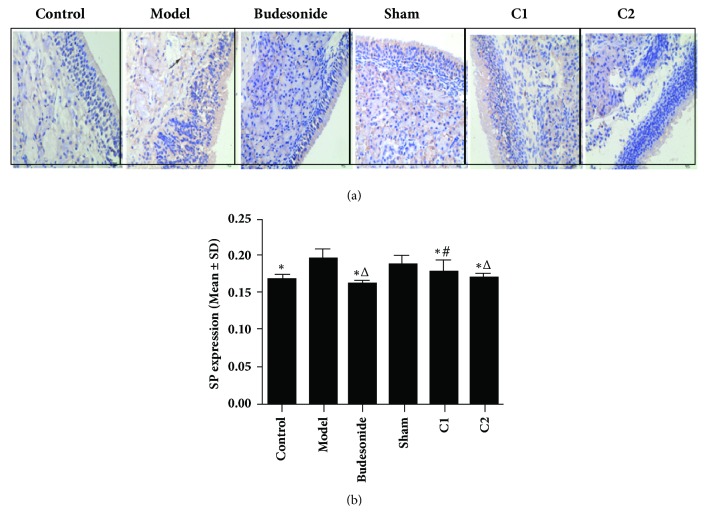
**Effects of CIAA on SP expression.** The experimental protocol (essentially the same as in Figure 1) and measurement of SP expression have been detailed in Materials and Methods. (a) IHC image; (b) quantification. *∗P*<0.01 versus the model group. ^△^*P*<0.01 versus the sham group. ^#^*P*<0.05 versus the C1 group. Scale bar for panel (a) = 50 *μ*m.

In the immunohistochemical (IHC) analysis (Figure 4), the expression of SP in the model group showed significant difference from the C1, C2, and Budesonide groups (*P *< 0.01). The expression was lower in the Budesonide group than in the C1 groups (*P* < 0.05; Figures 4(a) and 4(b)), whereas the C2 group showed no significant difference with the Budesonide group (*P *> 0.05). The expression in the C2 group was slightly lower than that in C1.

### 3.6. Effects of CIAA on TLR2 and TLR4 Expression

**Figure 5:**
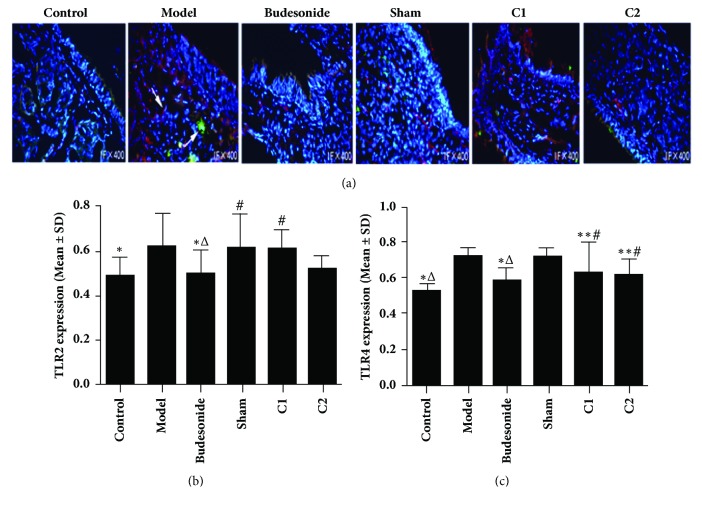
**Effects of CIAA on TLR2 and TLR4 expression.** The experimental protocol including fluorescence assays has been described in Materials and Methods. (a) Representative photographs showing TLR2 (green) and TLR4 (red) are shown. Nuclei were stained with DAPI (blue). The data are presented as mean ± SD. (b) TLR2 quantification: *∗*P<0.05 versus the model group; ^△^P<0.05 versus the sham group; ^#^P<0.05 versus Budesonide group. (c) TLR4 quantification: *∗*P<0.01 versus the model group; *∗∗*P<0.05 versus the model group; ^△^P<0.01 versus the sham group; ^#^P < 0.05 versus the sham group.

In our immunofluorescence analysis (Figure 5), we could not detect any significant difference in the expression of TLR2 in the nasal mucosa among the C1, C2, model, and sham groups (*P*>0.05). However, the TLR2 expression was lower in the Budesonide group than in the model, sham, and C1 groups (*P*<0.05). We also did not observe any significant difference in the expression of TLR2 in the Budesonide group, compared with the C2 group (*P *> 0.05). TLR4 expression in the nasal mucosa also showed no significant difference among the C1, C2, and Budesonide groups (*P *> 0.05) but was significantly lower in the Budesonide group compared to model and sham groups (*P *< 0.01; Figures 5(a)–5(c)). TLR4 expression was also lower in the C1 and C2 groups than in the model and sham groups (*P *< 0.05). Lastly, TLR4 in the model group showed no significant difference compared with the sham group (*P *> 0.05).

### 3.7. Effects of CIAA on the Cytosolic Expression of TLR2 and TLR4

**Figure 6:**
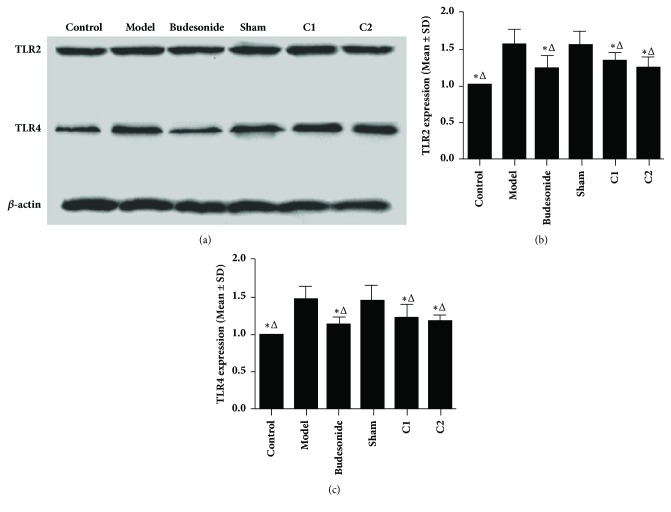
**Effects of CIAA on the cytosolic expression of TLR2 and TLR4. **(a) Representative Western blot images showing cytosolic expression of TLR2 and TLR4 in the indicated groups, as described in Materials and Methods. Actin serves as an internal control. (b) TLR2: *∗P *< 0.01 versus the model group; ^△^*P *< 0.01 versus the sham group. (c) TLR4: *∗P *< 0.01 versus the model group; ^△^*P*<0.01 versus the sham group.

In Western blot analysis of fractionated cell extracts, we detected the cytosolic expression of TLR2 and TLR4 in the nasal mucosal cells, which showed no significant difference among the C1, C2, and Budesonide groups (*P *> 0.05), but both were significantly lower in the C1, C2, and Budesonide groups than in the model or sham group (*P *< 0.01; Figures 6(a)–6(c)). Furthermore, the expression of TLR2 and TLR4 in the sham group showed no significant difference compared with the model group (*P *> 0.05).

## 4. Discussion

CIAA is based on the theory and practice of traditional acupuncture, which can extend the sensation and effect of needling because of the persistent stimulus to acupoints by the embedding catgut [14, 25]. Our results, presented here, reveal that CIAA has significant inhibitory effects on allergic inflammation, especially in inhibiting nasal symptoms and the degranulation rates of mast cells in nasal mucosa and the related proinflammatory cytokines, which is consistent with previous studies of ours and others on AR [25, 26]. Our results clearly showed that the Th2 cytokine (IL-4) and sIgE levels significantly decreased after CIAA treatment; in contrast, the Th1 cytokine (IFN-*γ*) significantly increased, which is in agreement with recent evidence of significant increase in IFN-*γ* and decrease in IL-4 [27, 28]. In addition, we found significant decrease of SP expression following CIAA treatment, which is also in agreement with several published studies [11, 15, 29]. Finally, in conditions of nasal hypersensitivity and compromised mucosal integrity, the expression levels of TLR2 and TLR4 significantly increased in the model group. However, CIAA is considered an activating stimulus to the tissue, mainly caused by needle injury and catgut homologous protein, which significantly downregulated TLR2 and TLR4 expression.

In China, CIAA has been particularly effective in treating chronic diseases and used for centuries to treat AR. While the majority of studies has focused on the anti-inflammatory mechanisms of acupuncture research, few have investigated the immunomodulatory effects of CIAA obtained from rats. In this study, we addressed the immunomodulatory effects of CIAA on AR, providing a basis for further clinical applications of CIAA on treating allergic diseases. In addition, our results demonstrate a potentially beneficial regimen in the treatment of AR.

The exact pathological mechanism of AR is currently not fully understood. Accumulating evidence has shown that neuroimmune abnormalities play important roles in the development of AR [30]. Excessive activation of T-helper 2 (Th2) and mast cell-mediated inflammatory reactions play a central role in AR. Specifically, several studies have shown that the Th2 cytokines, such as IL-4, are downregulated by CIAA or acupuncture [31, 32] and that the Th1 cytokines, such as IFN-*γ*, are also regulated [31, 33, 34]. Yet another study reported a reduction of IgE concentrations in the blood [35]; in this study, IL-4 levels significantly decreased after weeks of CIAA, and so did sIgE levels, while IFN-*γ* levels were significantly upregulated. These results support the proposal that a shift from Th2 to Th1 occurs after CIAA treatment, especially around two weeks.

In addition to the above-mentioned immune cell activation, neurogenic inflammation, such as SP expression in nasal mucosa, decreased after CIAA, which is in accordance with previous studies [11]. Our results (Figure 4) showed a trend in which the C2 group is lower than the C1 group in SP expression. This apparent effect of CIAA can be attributed to the persistent stimulus to acupoints by the embedding catgut. It is now well established that substance P plays an essential role in neurogenic inflammation in the nasal mucosa [36, 37]. SP is released from sensory nerves via axonal reflex, leading to vasodilation and plasma exudation to promote AR symptoms. However, the early-phase allergic response is mainly caused by the chemical mediators secreted from mast cells and basophils. Moreover, the chemical mediators stimulate nasal sensory neurons to release many neuropeptides, such as SP; histamine, a strong neurotransmitter, is also released in allergic inflammatory processes [38–41]. Overall, an interactive role between cytokines and neuropeptides underlies such allergic responses. Together, these results lead us to conclude that CIAA attenuates the development of nasal neurogenic and cytokine-mediated inflammation and eventually inhibits AR development.

Additionally, expression of Toll-like receptors, TLR2 and TLR4, which are upstream effectors of signaling pathways, decreased after CIAA. Our trend shows that the C2 group was lower than C1 in TLR2 and TLR4 expression, likely associated with the persistent stimulus of embedding catgut. A number of studies have identified associations between Toll-like receptor genes and AR [42]. In particular, TLR2 and TLR4, which are expressed on the surfaces of diverse immune cells, including dendritic cells, macrophages, and B- and T-lymphocytes [43], can modify cellular immune response and alter the host's susceptibility to disease [44].

There were some limitations associated with the current study, in part because all possible ranges of variables were not investigated. First, we did not examine other neuropeptides such as CGRP and VIP. Second, the exact mechanisms underlying the observed improvement in the model of AR through CIAA administration are likely to be more complex and remain to be elucidated. Third, although the C2 group showed the tendency to be lower than C1 in inflammatory readouts, the difference is not remarkable and its significance is unclear. Perhaps, if we provide a second CIAA on the rats, the effects of CIAA may be different from the present study. Future studies, incorporating more extensive experimental designs, complemented by* in vitro* studies, should provide a mechanistic basis for improvement of AR by CIAA treatment.

## 5. Conclusion

Based on the collective results, we conclude that CIAA at the Yingxiang (LI20) and Zusanli (ST36) acupoints effectively reduced allergic symptoms and inflammatory parameters in the rat model of AR. We suggest that CIAA treatment is potentially an alternative therapeutic modality in AR.

## Figures and Tables

**Table 1:** Serum levels of sIgE, IFN-*γ*, and IL-4 (mean ± SD).

Group	sIgE (ng/mL)	IFN-*γ* (mg/mL)	IL-4 (pg/mL)
Control	72.65±9.17	0.59±0.06	10.74±3.05
Model	124.92±15.56^#^	0.29±0.07^△^	16.04±3.81*∗*
Budesonide	126.34±13.20^#^	0.27±0.05^△^	16.75±5.01*∗*
Sham	127.06±14.20^#^	0.26±0.06^△^	16.90±2.75*∗*
C1	129.20±11.12^#^	0.28±0.06^△^	17.32±3.05*∗*
C2	127.49±12.51^#^	0.25±0.05^△^	17.18±2.98*∗*

Note: serum levels were measured as described under Methods. *∗P *< 0.01 versus the control group; ^△^*P *< 0.01 versus the control group; ^#^*P *< 0.01 versus the control group.

## Data Availability

Please contact author for any additional data requests.
